# Whole-genome sequence of the *Halobacillus naozhouensis* type strain KACC 21980

**DOI:** 10.1128/MRA.00570-23

**Published:** 2023-10-24

**Authors:** Hyorim Choi, Seunghwan Kim, Yiseul Kim, Jun Heo

**Affiliations:** 1 Agricultural Microbiology Division, National Institute of Agricultural Sciences, Rural Development Administration, Wanju-gun, Jeollabuk-do, South Korea; 2 Division of Biotechnology, Chonbuk National University, Iksan-si, Jeollabuk-do, South Korea; Montana State University, Bozeman, Montana, USA

**Keywords:** *Halobacillus naozhouensis*, type strain, genome, Pacbio, Illumina

## Abstract

We present the whole-genome sequence of *Halobacillus naozhouensis* Korean Agricultural Culture Collection (KACC) 21980^T^, isolated from China by Chen et al.. The genome of *Halobacillus naozhouensis* KACC 21980^T^ comprises a circular chromosome (4.2 Mb) and one plasmid (17 kb). It includes a total of 4,168 predicted coding genes.

## ANNOUNCEMENT

The genus *Halobacillus* was initially classified in 1996 (1). These strains are characterized as aerobic, Gram-positive, motile with flagella, endospore-forming, rod-shaped, and halophilic bacteria. Since its first report in 1996, the genus comprises 22 validly published species listed in the List of Prokaryotic Names with Standing in Nomenclature. Among them, *Halobacillus naozhouensis* was first documented by Chen et al. in China (2), having been isolated from homogenates of a sea anemone (*Anthopleura xanthogrammica*) on a tidal flat at Naozhou Island, situated at the edge of the South China Sea (2). However, until now, there have been no further reports on *H. naozhouensis*. As part of the genome database construction project deposited in Korean Agricultural Culture Collection (KACC), we conducted a genome analysis.

Here, we analyze and report the whole-genome sequence of *Halobacillus naozhouensis* KACC 21980^T^. The strain was obtained from KACC and cultured on marine agar medium (KisanBio, Seoul, Korea) at a pH of 7.5 and 28℃ for 2 days under aerobic conditions. Genomic DNA (gDNA) for PacBio and Illumina sequencing was extracted using the Qiagen MagAttract HMW DNA kit (Qiagen, Hilden, Germany), following the manufacturer’s instructions. Whole-genome sequencing was performed using the Illumina NovaSeq 6000 (Illumina, San Diego, USA) and PacBio Sequel IIe (Pacific Biosciences, Quebec, Canada) platforms. The gDNA libraries were generated using the TruSeq Nano DNA High Throughput Library Prep Kit (Illumina). Default settings were used for all software unless otherwise indicated. For the template of PacBio SMRTbell prep kit v.3.0, a total of 10-µL libraries were prepared and analyzed with Sequel II Bind Kit v.3.2 and Int Ctrl v.3.2. Sequencing was carried out using Sequel II Sequencing Kit v.2.0 and SMRT cell 8M trays. HiFi reads were produced from the PacBio Sequel IIe system. Microbial assembly was performed with SMRTlink 11.0.0.146107, based on the Hierarchical Genome Assembly Process (3). Following the generation of short and long reads, they were quality-filtered and adapter-trimmed using Trimmomatic v.0.38. Then, the assembly based on long-read data was refined with Illumina reads to correct errors and enhance accuracy using Pilon v.1.21 (4). Genome annotation was conducted using the National Center for Biotechnology Information Prokaryotic Genome Annotation Pipeline v.6.4 (5). All tools were employed with default parameters, unless otherwise specified. Illumina Novaseq 6000 and PacBio Sequel IIe sequencing produced 20,430,428 read pairs (2 × 150 bp) and 138,199 long reads (1,229,020,427 bp; 10,112 *N*_50_), respectively. The genome of *H. naozhouensis* KACC 21980^T^ consists of a single circular chromosome (4,237,314 bp with a G+C content of 41.5%, sequence depth 287.1×) and one circular plasmid (17,876 bp with a G+C content of 38.0%, sequence depth 897.9×) (Table 1). The contig assembly includes 4,168 total coding sequences, 21 rRNA genes, 68 tRNA genes, 4 non-coding RNA genes, and 51 pseudogenes.

**TABLE 1:** Genome statistics of the *Halobacillus naozhouensis* KACC 21980^T^

Genome statistics	*Halobacillus naozhouensis* KACC 21980^T^
GenBank accession no.	CP121671–CP121672
Genome size (bp)	
Chromosome	4,219,438 (CP121671)
Plasmid	17,876 (CP121672)
Sequence depth	
Chromosome	287.1
Plasmid	897.9
G+C content (%)	41.5
Total number of genes	4,219
Protein coding genes (CDS)	4,168
rRNA genes (5S, 16S, 23S)	7, 7, 7
tRNA genes	68
ncRNA genes	4
Pseudogenes	51

## Data Availability

The complete genome sequence of *Halobacillus naozhouensis* Korean Agricultural Culture Collection 21980^T^ has been deposited in National Center for Biotechnology Information (NCBI) GenBank under BioProject PRJNA952362 with BioSample accession number SAMN34073859 and GenBank accession numbers CP121671–CP121672. Raw Illumina and PacBio reads are available under NCBI’s SRA accession numbers SRR24204045 and SRR24204044, respectively.
